# Acute Appendicitis: Clinical Clues and Conundrums Related to the Greatest Misses

**DOI:** 10.7759/cureus.8051

**Published:** 2020-05-11

**Authors:** Ricki Brown-Forestiere, Anthony Furiato, Nikolas P Foresteire, John S Kashani, Abdul Waheed

**Affiliations:** 1 Emergency Medicine, Brandon Regional Hospital/Hospital Corporation of America (HCA)-University of South Florida (USF) Consortium, Brandon, USA; 2 Emergency Medicine, Brandon Regional Hospital, Brandon, USA; 3 Emergency Medicine, Lake Erie College of Osteopathic Medicine, Bradenton, USA; 4 Emergency Medicine, St. Joseph's University Medical Center, Paterson, USA; 5 Surgery, Brandon Regional Hospital, Brandon, USA

**Keywords:** right lower quadrant pain, periumbilical pain, mcburney's, rovsing, psoas, missed, misdiagnosed, acute appendicitis

## Abstract

Introduction

In the United States (US), appendicitis is the most common acute abdominal emergency requiring surgery. Patients with appendicitis continue to display a complex and atypical range of clinical manifestations, providing a subsequent high risk for emergency physicians to miss acute abdominal pathology on a patient’s initial visits. Due to the risk of potential perforation, the proper and timely clinical identification of acute appendicitis is vital. The current study aims to identify clinical characteristics that could be useful in identifying patients at risk for having acute appendicitis that was misdiagnosed on their initial visits.

Methods

Medical charts consisting of patients between the ages of 19 and 55 years on their second visit were flagged and reviewed by the emergency department quality assurance (EDQA) committee. The retrospective chart review included patients who presented to the emergency department (ED) with the chief complaint of an abdominal-related complaint, were discharged, returned within 72 hours, and were diagnosed with a pathologically confirmed appendicitis. All patients were managed operatively, with pathology results reviewed for evidence of acute appendicitis. Those with confirmed pathologic appendicitis upon return were considered to have a “misdiagnosis.” Any patients managed nonoperatively and those with negative pathology were excluded from the study and considered not to have appendicitis.

Results

Fifty-five patients were identified through the EDQA committee from May 2011 to January 2014. After exclusion criteria were applied, 18 patients met the inclusion criteria for this study (7 males, 11 females). The mean age was 36.2 (range: 19-55). The most common presenting complaint on the initial visit was pain in the epigastric region of the abdomen (50%, n = 9). Twenty-two percent (n = 4) of patients had pain in the right lower quadrant documented in the physician’s note on the initial visit and 83% (n = 15) had right lower quadrant pain documented on the second visit. Two patients (11%) did not have right lower quadrant tenderness on either visit. The most common discharge diagnosis on the initial visit was undifferentiated abdominal pain (50%), followed by gastritis (28%). Opioid pain medication was administered or prescribed to 39% (n = 7) of the patients. The average return time was 23.9 hours.

Conclusion

The administration of opioid pain medication is associated with many of the return visits to the emergency department for missed appendicitis. Finally, discharge diagnosis and planning are imperative, as detailed early appendicitis instructions or extended ED observation can include more cases and decrease litigation risk.

## Introduction

Up to 300,000 appendectomies are performed each year in the US. With an average lifetime risk of up to 25%, it is the most common abdominal surgical emergency [1]. Most of the published medical literature of the misdiagnosis of appendicitis focuses on the pediatric, female, and geriatric populations [2-3]. Challenges to the diagnosis of appendicitis in these populations may stem from the reluctance to subject a pediatric patient to ionizing radiation, reproductive tract alternatives to diagnosis in the childbearing aged female, and atypical presentations in the geriatric population [4-5].

Classically, the presentation of acute appendicitis is described as periumbilical or upper abdominal pain that gradually transitions or radiates to the right lower quadrant [6]. However, this presentation may only be present in approximately 50% of patients with acute appendicitis. Case reports have even cited acute right lower quadrant pain and flank pain as the initial presenting symptom for acute appendicitis. A myriad of symptoms comprises the initial presentation to the emergency department, including bowel irregularities, anorexia, nausea, and fever [6].

The initial exam also remains variable, ranging from a benign soft abdomen to peritoneal signs. The classic exam signs of McBurney’s point tenderness, Rovsing’s sign, and Psoas sign remain notoriously nonspecific and do not offer a significant increase to the post-test probability [7]. Multiple clinical tools and laboratory tests are available to assess suspected appendicitis for decreasing the utilization of computed tomography (CT) scans and the subsequent exposure to ionizing radiation. Clinical scoring tools have also been formulated, including the Alvarado score, Lintula score, and Raja Isteri Pengiran Anak Saleha Appendicitis (RIPASA) score [8].

Unfortunately, the clinical decision rule suffered from poor sensitivities and did not provide an adequate “rule out” strength for routine use in the emergency department [8]. More recent studies have shown that using a scoring system as a guide for pre-test probability combined with serum inflammatory markers, such as C-reactive protein (CRP) and bilirubin, may provide better sensitivities but remain to be clinically validated [8]. The current study identifies potential useful historical and clinical characteristics in the identification of patients with acute appendicitis that were misdiagnosed during their initial visits.

## Materials and methods

Our study was a qualitative, retrospective analysis of the collected data on all patients who returned to our emergency department within 72 hours following an initial visit for abdominal pain. They were discharged home on the initial visit with a diagnosis other than acute appendicitis and were ultimately diagnosed with pathology proven acute appendicitis on the second visit. Data collection was over three years from May 2011 to January 2014. The study was performed in Paterson, NJ, at St. Joseph’s Regional Medical Center located in the tri-state area of New York City, with a busy urban emergency department with an annual volume of 170,000 visits per year.

Patients were seen in the emergency department by residents of emergency medicine, internal medicine, general surgery, family medicine, and board-certified emergency medicine attendees. The hospital institutional review board (IRB) approved this study before the initiation of data collection. Patients were identified by an EDQA committee and reviewed for clinical appropriateness. All patient data, including age, sex, appendix pathology results (including perforation), time of return visit and opioid medication administration on the initial visit, presenting chief complaint, abdominal exam on all visits, and initial discharge diagnosis were obtained through the emergency department and hospital computerized record system, respectively, MEDHOST 2013 (MEDHOST Co., Franklin, TN) and Soarian Clinicals 2014 (Cerner Corp., North Kansas City, MO).

Data collected were entered into an Excel spreadsheet (Microsoft Excel 2010, Microsoft Corporation, Redmond, WA). All patients’ identifying data were stored on a secure password-protected computer. Patient data were de-identified with an alphanumeric code and, subsequently, password protected again. All patients underwent either conventional or laparoscopic appendectomy. Patients who had normal pathology of the appendix were excluded. Data were analyzed using Microsoft Excel 2010. Descriptive statistics were provided as a proportion (percent, %). Variables with significant variations were presented as median with interquartile range. Standard calculations were completed using basic statistical methods.

## Results

Between May 2011 and January 2014, 55 patients were identified through the EDQA committee for review. After exclusion criteria were applied, 18 patients were included in this study. Of those 18 patients, seven (38%) were male and 11 (62%) were female (Tables 1-2).

**Table 1:** Key clinical results RLQ = Right lower quadrant; N = Number of patients

Factors	Percentage (%) of males (N=7):	Percentage (%) of females (N=11):	Total population percentage (N=18): (%)
Non-Tender Initial Encounter	85.7	72.7	77.8
RLQ Initial Encounter	14.3	27.3	22.1
Epigastric Initial Encounter	57.1	45.5	49.9
RLQ 2^nd^ Encounter	100	72.7	83.2
Opioid Pain Medication	14.2	54.5	38.8
Perforation Present	57.1	18.1	33.3

**Table 2:** Most Common Discharge Diagnoses *Male sample population excluded from ovarian cyst discharge diagnosis due to the anatomical lack of ovaries; N = Number of patients

Initial Discharge Diagnosis	Percentage (%) of males (n=7):	Percentage (%) of females (n=11):	Total population percentage (N=18): (%)
Abdominal Pain	42.9	54.5	50
Gastritis	28.6	27.3	27.8
Urinary Tract Infection	0.0	18.2	11.1
Constipation	14.3	0.0	5.5
Ovarian Cyst	*	9.1	5.5

The mean age was 36.2 (range: 19-55). The most common presenting complaint on the initial visit was a pain in the epigastric region of the abdomen (50%, n = 9). Twenty-two percent (n = 4) of patients had pain in the right lower quadrant documented in the physician’s note on the initial visit and 83% (n = 15) had right lower quadrant pain documented in the second visit. Two patients (11%) did not have right lower quadrant tenderness on either visit. (Tables 2-3).

**Table 3:** Patient clinical data N = Never; yr = Years; hrs = Hours; F = Female; M = Male; LUQ = Left upper quadrant; LLQ = Left lower quadrant; RLQ = Right lower quadrant; Y = Yes; UTI = Urinary tract infection; D/C DX = Discharge diagnosis

Patient #	Perforated	Age (yr)	Return Time (hrs)	Gender	Opioids	Presenting Symp	RLQ 1	RLQ 2	D/C DX
1	N	19	17	F	N	Vomiting, LUQ/LLQ pain	N	Y	Ovarian Cyst
2	N	21	37	M	N	LUQ pain	Y	Y	Abdominal pain
3	N	52	57	F	Y	Epigastric pain, vomiting	N	N	Abdominal pain
4	N	41	11	F	Y	Vomiting, abdominal pain	N	Y	UTI
5	N	25	12	M	N	Epigastric pain	N	Y	Gastritis
6	Y	52	48	F	N	Epigastric pain, b/l flank pain	N	Y	UTI, Gastritis
7	Y	54	12	M	N	Epigastric pain	N	Y	Constipation
8	N	26	11	M	N	Periumb pain	N	Y	Abdominal pain
9	N	30	16	F	Y	RLQ/R flank pain	Y	Y	Abd pain, Kidney stones
10	N	36	34	F	Y	Epigastric pain	N	Y	Abdominal pain
11	N	55	24	F	Y	Abdominal pain, nausea, vomiting	Y	Y	Gastritis
12	Y	28	16	M	N	Epigastric pain, vomiting	N	Y	Gastritis
13	N	30	17	F	N	Epigastric pain	N	Y	Gastritis
14	N	24	15	F	N	Epigastric pain	N	N	Gastritis
15	Y	51	24	M	Y	Epigastric pain, nausea, vomiting	N	Y	Abdominal pain
16	N	31	16	F	N	Suprapubic pain	N	N	Abdominal pain
17	Y	27	48	M	N	Periumbilical pain	N	Y	Abdominal pain
18	Y	50	15	F	Y	Lower abd pain	Y	Y	Abdominal pain, cervicitis

The most common discharge diagnosis on the initial visit was undifferentiated abdominal pain (50%), followed by gastritis (28%). Opioid pain medication was administered or prescribed to 39% (n = 7) of the patients. The average return time was 23.9 hours. The total number of patients who had histologic evidence of perforation was six (33%) (Table 3).

## Discussion

The diagnosis of acute appendicitis remains a difficult diagnosis to make secondary to multiple factors, including the inherent progression of the disease process and the substantial heterogeneity in the patient population [9]. Most of the published medical literature of missed appendicitis concerns the pediatric population, females of childbearing age, and the geriatric population [10-13]. Classically, the appendicitis presentation is an upper abdominal or periumbilical pain that gradually migrates to the right lower quadrant over 24-48 hours [14-17].

**Figure 1:**
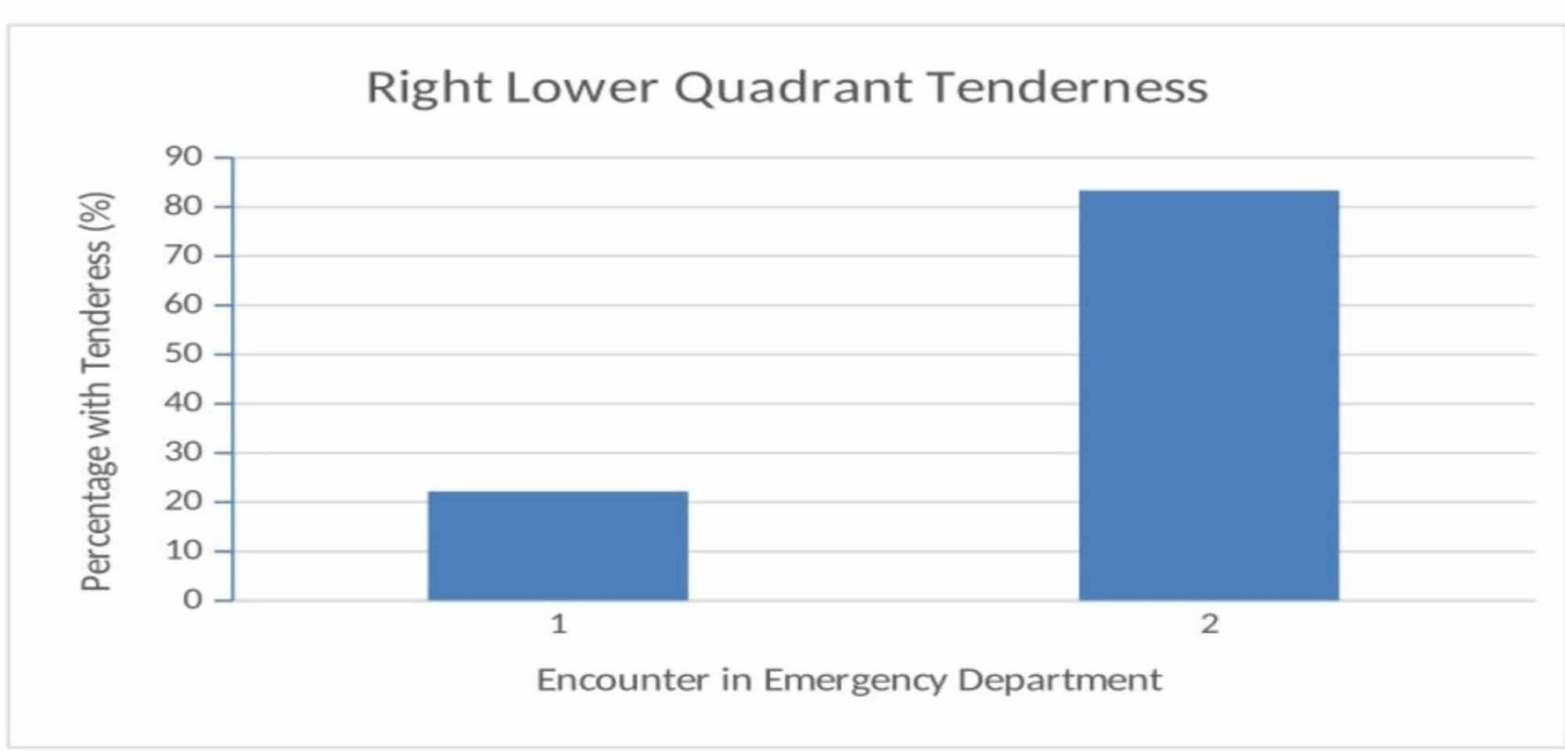
Abdominal tenderness, encounter comparison % = Percentage of patients

This study has shown that this is indeed the case with the main presenting chief complaint as epigastric pain with a right lower quadrant that is nominally tender in 22% of patients on the initial visit, followed by a sharp increase to 83% of patients having right lower quadrant tenderness on the second visit exam (Figure 1). This illustrates that in this patient population, classic disease progression should be critically assessed and formulated into a working plan with careful discharge planning for possible early appendicitis. It also highlights the importance of extended observation in the emergency department in a select group of patients with repeat abdominal exams.

Of note, two patients in this study did not have right lower quadrant tenderness on either visit. Multiple case studies have reported that tenderness on the exam may correlate to the degree of inflammation of the appendix and surrounding tissue and the location of the appendix [11-12]. Retrocecal appendices may cause less localized pain while a pelvic appendix may cause urinary symptoms or diarrhea [3]. These cases appear to be a minority of presentations.

Additionally, the perforation of the appendix has historically led to increased morbidity and mortality, with a mortality rise from 1% to 3% if perforation occurs [3,16]. Papandria et al. report a 28.8% perforation rate if surgical management is completed on the day of presentation [3]. The risk of perforation increases to 33.3% by Day 2 and will then dramatically rise to 78.8% by one week [3]. In the current study, the return time was within 72 hours, with the majority of patients within 48 hours. The perforation rate was 33%, which correlates with the aforementioned study’s results. This again highlights the importance of early recognition of appendicitis.

Also, historically, the diagnosis of appendicitis in females is more difficult due to gynecologic considerations of urinary tract infection, pelvic inflammatory disease, ovarian cysts, and torsion, making them more likely to have a perforation when the diagnosis of acute appendicitis is made [6]. Interestingly, our study concluded that of the patients who had evidence of perforation, most were male (67%). Rothrock et al. reported a lower incidence of abdominal tenderness on the exam in females, leading to a search for other potential causes of abdominal pain [6]. In our study, females account for 50% of the nontender abdominal exams on the initial visit. Of the two patients mentioned previously who had nontender abdomens on both initial and subsequent visits, both were female. Guss et al. also report similar findings with a longer time to operative interventions for females versus their male counterparts, but no significant increase in the incidence of perforation was substantiated [17].

Moreover, the discharge diagnosis and planning for any patient in the emergency department is crucially vital for patient safety and the continuance of care. This study has shown that of the patients that were discharged home on their initial encounter, the most common diagnosis was abdominal pain followed by gastritis. Rusnak et al. reported in 1994 that the most common diagnoses of missed appendicitis that went to litigation were gastroenteritis, abdominal pain, peptic ulcer disease, and urinary tract infection [8]. This leaves a potential area for the improvement of practice by broadening the subset of patients who are discharged from the emergency department with early appendicitis precautions and instructions, as the same diagnoses are used in this study at discharge.

Furthermore, Rusnak et al. report on the use of narcotic pain medication use in cases that have gone to litigation. From his retrospective review of malpractice claims, narcotic pain medication administration was administered before discharge in 56% of the cases [8]. They advocate for extended observation or surgical consultation before discharge for any patient given narcotics for abdominal pain without a definite diagnosis. In our study, 39% of patients were given opioid pain medication prior to discharge, leaving another subset of the patients who may have benefitted by extended observation or surgical consultation.

## Conclusions

To our knowledge, no other study published in the medical literature has been able to compare initial and subsequent visits of patients with pathology-proven appendicitis. Besides, we were able to provide a time estimate for a return visit that ultimately led to the diagnosis of acute appendicitis. Migration of pain from the epigastric/periumbilical area to the right lower quadrant, although considered classic, is only seen in up to 50% of patients presenting with acute appendicitis. Opioid pain medication is associated with many of the return visits to the emergency department for missed appendicitis. Finally, discharge diagnosis and planning is imperative, as detailed early appendicitis instructions or extended ED observation can include more cases and decrease litigation risk.
